# Spatially-localized bench-top X-ray scattering reveals tissue-specific microfibril orientation in Moso bamboo

**DOI:** 10.1186/s13007-016-0155-1

**Published:** 2017-01-09

**Authors:** Patrik Ahvenainen, Patrick G. Dixon, Aki Kallonen, Heikki Suhonen, Lorna J. Gibson, Kirsi Svedström

**Affiliations:** 1 Department of Physics, University of Helsinki, P.O. Box 64, 00014 Helsinki, Finland; 2 Department of Materials Science and Engineering, Massachusetts Institute of Technology, 77 Massachusetts Ave, 02139 Cambridge, MA USA

**Keywords:** Bamboo parenchyma, *Phyllostachys edulis*, Wide-angle X-ray scattering, X-ray microtomography, Microfibril angle, Spatially-localized scattering, Diffraction-contrast tomography

## Abstract

**Background:**

Biological materials have a complex, hierarchical structure, with vital structural features present at all size scales, from the nanoscale to the macroscale. A method that can connect information at multiple length scales has great potential to reveal novel information. This article presents one such method with an application to the bamboo culm wall. Moso (*Phyllostachys edulis*) bamboo is a commercially important bamboo species. At the cellular level, bamboo culm wall consists of vascular bundles embedded in a parenchyma cell tissue matrix. The microfibril angle (MFA) in the bamboo cell wall is related to its macroscopic longitudinal stiffness and strength and can be determined at the nanoscale with wide-angle X-ray scattering (WAXS). Combining WAXS with X-ray microtomography (XMT) allows tissue-specific study of the bamboo culm without invasive chemical treatment.

**Results:**

The scattering contribution of the fiber and parenchyma cells were separated with spatially-localized WAXS. The fiber component was dominated by a high degree of orientation corresponding to small MFAs (mean MFA 11°). The parenchyma component showed significantly lower degree of orientation with a maximum at larger angles (mean MFA 65°). The fiber ratio, the volume of cell wall in the fibers relative to the overall volume of cell wall, was determined by fitting the scattering intensities with these two components. The fiber ratio was also determined from the XMT data and similar fiber ratios were obtained from the two methods, one connected to the cellular level and one to the nanoscale. X-ray diffraction tomography was also done to study the differences in microfibril orientation between fibers and the parenchyma and further connect the microscale to the nanoscale.

**Conclusions:**

The spatially-localized WAXS yields biologically relevant, tissue-specific information. With the custom-made bench-top set-up presented, diffraction contrast information can be obtained from plant tissue (1) from regions-of-interest, (2) as a function of distance (line scan), or (3) with two-dimensional or three-dimensional tomography. This nanoscale information is connected to the cellular level features.

## Background

Biological materials have a hierarchical structure that connects their features at different length scales, from the atomic to the macroscale, to their function and form. A method that allows connecting the cellular level information to the nanoscale can open up new levels of understanding that are not possible without that vital connection. Spatially-localized X-ray scattering is an accessible method to bridge the nanoscale to the cellular structure. In this article a bench-top set-up [1] combining X-ray scattering and microtomography is used to provide novel structural information from bamboo culm wall.

*Phyllostachys edulis* is economically the most important bamboo species in the world in the multi-billion euro bamboo industry [2]. Also known as *P. pubescens* [3] (henceforth referred to as Moso), it is a large and woody bamboo species native to China. While it is also known for its edible shoots, the mature bamboo culm is used for its excellent structural properties.

Although a member of the grass family (*Poaceae*), bamboo is often used as a timber-like construction material. It is known for its exceptionally fast growth (over 1 m in 24 h [4]), high strength and stiffness [5], and excellent fracture toughness [6]. Bamboo is a renewable and sustainable material that has potential in structural bamboo products analogous to wood products such as plywood, and oriented strand board [2, 7, 8], fiber-reinforced composites [9] and in many other products, such as furniture, handicrafts, scaffolding, flooring and construction [2].

Unlike wood, bamboo, as a member of the grass family, does not produce secondary growth. The primary shoots emerge and expand to their final height during one rainy season [2]. The bamboo tissue then typically matures over the following 4–5 years [10]. Structurally bamboo can be seen as a composite material where the vascular bundles are embedded in a matrix of parenchyma cells [11]. The bamboo culm is functionally graded and highly heterogeneous [12, 13]. In the longitudinal direction, the bamboo culm is separated by nodes into several internodes. In one internode section the parenchyma and vascular bundles are well aligned with the longitudinal axis of the culm [14]. In the radial direction, the density of bamboo and the proportion of vascular bundles increase from the interior to the exterior [14]. The vascular bundle structure varies by bamboo species, but contains always fibers, vessels and other cells [2].

The bamboo culm cell wall consists mainly of cellulose, hemicelluloses and lignin [2]. Cellulose is present in the cell wall as long microfibrils with alternating amorphous and crystalline regions. The angle that the microfibrils form with the longitudinal axis of the cell is called the microfibril angle (MFA). The bamboo cell wall, both in the parenchyma and fiber cells, is separated into multiple layers with alternating microfibril orientation [2]. The longitudinal axes of the parenchyma and fiber cells are parallel to the longitudinal axis of the culm wall.

Cellulose microfibril orientation in different bamboo tissue types has been studied with field emission scanning electron microscope (FESEM) by Crow and Murphy [15]. With chemical treatment and FESEM observations they reported varying MFAs in different cell wall layers in both fiber and parenchyma cells. The crystallinity of bamboo parenchyma cells has more recently been studied by Abe and Yano [16] who treated the bamboo culm chemically to separate microfibril aggregates from the parenchyma cells and the fiber cells.

In complex heterogeneous materials such as bamboo, the interpretation of the scattering data is complicated due to the presence of several cell types and cell wall layers. Thomas et al. [17] measured internode tissue of mature bamboo with WAXS and suggest that the azimuthal orientation distribution could be dissected in two components that originate either from different cell wall layers or from different cell types. The aim of the current study is to obtain tissue-specific scattering components and help explain their contribution also in scattering data that is not tissue-specific.

To the authors’ knowledge, this study is the first tissue-specific study of microfibril orientation in native bamboo using an in-house set-up. Because both of the X-ray methods used, WAXS and X-ray microtomography (XMT), are nondestructive and thus the sample does not require any invasive chemical treatment, the results are obtained from the bamboo cell in its natural^1^, albeit dried, state. The same set-up is also used here to present an X-ray diffraction tomography (XDT) measurement on Moso bamboo with different diffraction-dependent contrasts. The localized X-ray scattering (LXS) set-up used in this article has previously been presented with an application to micrometeorites [1, 18] and to compacted clays [19].

Most XDT experiments are carried out at synchrotrons [20–23] although the viability of the method has recently been shown with in-house experiments as well [1, 24]. However, so far, outside of synchrotrons, both LXS and XDT have scarcely been applied to plant and other biological materials.

The method presented here is shown to yield biologically significant results, both qualitative and quantitative, that are not possible without the combined information from XMT and WAXS. The method could be applied to other biologically relevant systems as well, such as archaeological plant samples, hypocotyl and roots of *Arabidopsis thaliana* and other grass plants, never-dried wood and reaction wood.

## Methods

### Samples

**Fig. 1:**
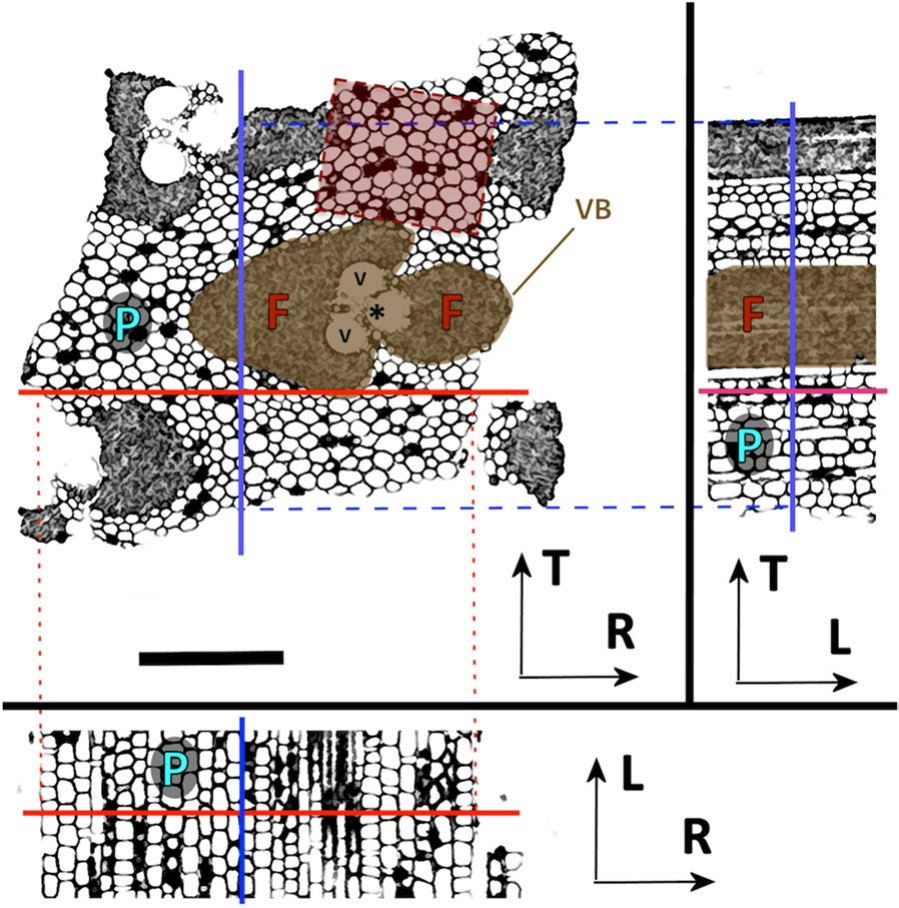
Various cross-sections of a Moso bamboo sample. (T–R) The bamboo culm wall pieces comprise of vascular bundles (VB) embedded in a parenchyma (P) matrix. One vascular bundle (VB, shaded with brown/gray) comprises of fibers (F), two metaxylem vessels (V) and the metaphloem (*). The *red*/*gray rectangle* shows the region-of-interest used for reconstructing parenchyma cell lumens. (T–L) The longitudinal axes of the fibers and parenchyma are parallel. (L–R) The aspect ratio is low in the parenchyma. *Scale bar* is 400 $\mu$m. The *arrows* indicate the orientation with respect to the bamboo culm wall: L = longitudinal, R = radial and T = tangential

Tangential slices (n = 8, radial thicknesses of 1.3–1.9 mm) were cut from a single internode section of Moso bamboo, half of them from the inner third of the culm wall (n = 4) and half of them from the outer third (n = 4). Outer samples do not contain the hard epidermal region, and inner samples do not contain the pithy terminal layer. For microtomography the slices were cut to a tangential width of approximately 1.5 mm so that the radial and tangential dimensions were approximately equal, as this geometry is more suitable for tomography. An example of the final cross-section of a bamboo sample is shown in Fig. 1, where also the different culm wall cell types are presented.

### Experimental

All LXS, XMT and XDT measurements were done with a custom-built combined X-ray microtomography–X-ray scattering set-up (set-up 1 [1]). Set-up 1 consists of two separate, independently operable, X-ray devices, which are described in detail in [1, 19, 25]. The two functionalities are connected by a shared sample manipulator stage, which allows the spatial alignment of the sample to be calibrated into XMT coordinates for the scattering modality.

The tomography functionality is provided by a custom-built high-resolution XMT scanner (Nanotom 180NF, GE Measurement and Control Solutions, Germany), which was built inside a lead-shielded room that allows more customization than a traditional radiation shielding cabinet. The XMT functionality is based on a transmission-type microfocus X-ray tube and a CMOS flat-panel detector (C7942SK-25, Hamamatsu Photonics, Japan). In the cone beam geometry optical magnification can be used to select the field of view and the scan resolution.

The pencil-beam scattering functionality is provided by a Mo-anode source ($\hbox {I}\mu \hbox {S}$, Incoatec GmbH, Germany) and a two-dimensional detector (Pilatus 1M, Dectris Ltd, Switzerland; maximum sample-to-detector distance 75 cm). The Mo-K$\alpha$ energy (17 keV) is selected using focusing Montel multilayer mirrors and the beam size and shape is adjusted with a variable divergence aperture and a vertical slit.

Beam alignment for set-up 1 with the X-ray microtomography coordinates was conducted using a small silver behenate particle, as in [1]. After the alignment, regions-of-interests (ROIs) for micro-diffraction could be selected directly from the tomographic reconstruction slice.

In addition to set-up 1, a conventional two-dimensional X-ray scattering set-up that is described elsewhere [26] (set-up 2) was used for X-ray scattering with a larger beam to obtain comparative bulk average information (average bamboo tissue).

#### X-ray microtomography

XMT was conducted either with a pixel size of $2.0\,\mu \hbox {m}$ resulting in a field of view of 2.3 mm (with a $2 \times 2$ pixel binning) or with a pixel size of $1.5\,\mu \hbox {m}$ and a field of view of 3.4 mm (without binning). At least 600 projection images were taken over the 360-degree scanning range. The X-ray tube was used with a $160/180\,\mu \hbox {A}$ tube current and an 80 kV acceleration voltage. To reduce noise, a total of 7 transmission images of 250 ms exposure each were averaged to produce one projection image.

**Fig. 2:**
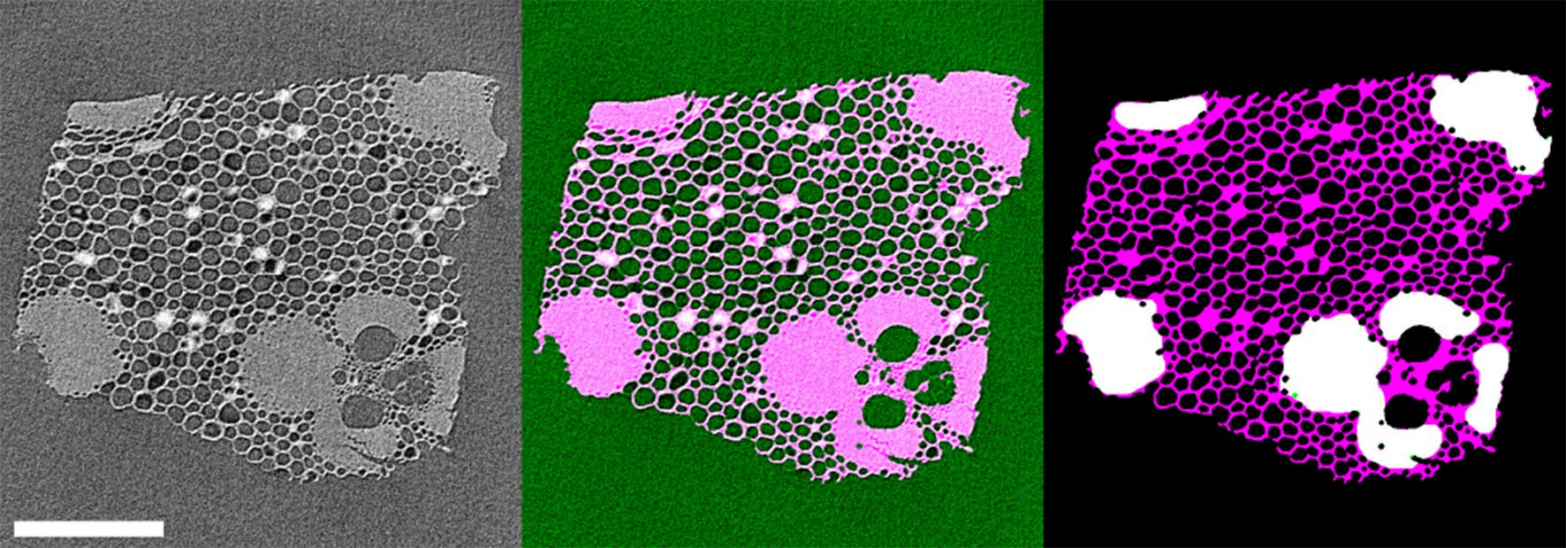
Tomographic reconstruction slice and binarization of Moso bamboo. (*Left*) Tomographic reconstruction, scale bar is $400\,\mu \hbox {m}$. (*Middle*) The binarized image overlayed on the reconstruction. The cell walls are shown in *magenta*/*light gray*. (*Right*) The cell walls separated by cell type: fibers (*white*) and others (*magenta*/*gray*)

Tomographic reconstruction slices were filtered with a non-linear diffusion filter before binarization in Matlab R2014a (Mathworks, USA). A morphological closing operation was used to close cell walls and a morphological opening to remove small objects. The fiber cell walls were selected by using a large median filter and using a large structuring element in the morphological opening of the image. An example of the binarization and cell type separation is shown in Fig. 2.

#### Wide-angle X-ray scattering

All scattering measurements were performed with perpendicular transmission geometry using a two-dimensional detector, with a measurement time of 30 min to obtain a sufficient signal-to-noise ratio^2^. Set-up 1 measurements were corrected for detector geometry and those of set-up 2 were corrected as in [11]^3^ prior to data analysis. Set-up 1 used Mo K$\alpha$ energy of 17.0 keV and set-up 2 used Cu K$\alpha$ energy of 8.0 keV. The data are thus presented in energy-independent units of the scattering vector length $q={4\pi \sin (\theta )}/{\lambda }$, where $\theta$ is half of the scattering angle $2\theta$ and $\lambda$ is the X-ray wavelength.

#### X-ray diffraction tomography

X-ray diffraction tomography was performed for one sample using a pencil beam geometry. A total of 31 rotation steps were taken over 180°. At each rotation step a 2.85-mm long line scan was completed with a step size of $150\,\mu \hbox {m}$ by measuring the scattering pattern for 30 s at each step, yielding a total duration of 5 h 10 min.

Two kinds of contrast were calculated from the 2D diffraction patterns: (1) degree of fibril orientation and (2) cellulose I content. These values were used as input for tomographic reconstruction done in Matlab.

To obtain the contribution of the degree of orientation, the difference was determined between the intensity of the 200-peak in the direction parallel to the preferred fiber orientation and in the direction perpendicular to it. Forty-degree sectors were chosen over the corresponding azimuthal angles. Radially the sectors extended from $q = 1.485$ Å$^{-1}$ to $q=1.555$ Å$^{-1}$.

To obtain the cellulose I contribution, the intensity value of the 200-peak perpendicular to the fiber orientation was chosen as this is a good indicator for cellulose I, regardless of the cell type.

Before reconstruction, bilinear interpolation was used to artificially increase both the number of translation and rotation steps with a factor of 2. After reconstruction a bilinear interpolation was also used to the diffraction tomography slices.

### Data analysis

#### Fiber ratio

Fiber ratio is determined here as the ratio of fiber cells to all cells. It was assessed both from the XMT and the WAXS data. From the tomographic reconstructions it was calculated as the cell wall volume of fiber cells relative to the total cell wall volume.

The azimuthal integrals from a WAXS line scan (Fig. 8) were fitted in Matlab with a two-component model using non-negative matrix factorization (NNMF). One component represents scattering from bamboo fibers and the other from other types of cells (mainly parenchyma cells, referred to as the parenchyma model from here on out). The fiber ratio from WAXS was obtained then from the azimuthal scattering intensities by taking the relative weight of the fiber model.

#### Aspect ratio of parenchyma cells

An aspect ratio (AR) was estimated for the parenchyma cells from the highest resolution tomographic reconstruction by first selecting a ROI consisting of only parenchyma cells. The maximum height ($H_{l}$, along the longitudinal axis of the culm) and maximum cross-sectional area ($A_{l}$) were calculated for each segmented parenchyma cell lumen (n = 1667). The cross-sectional cell shape was assumed to be roughly circular and the cell AR ($AR_{c}$) was approximated from the cell lumen dimensions as

$$\begin{aligned} AR_{c} = \frac{H_{l}}{\sqrt{A_{l} \frac{4}{\pi }}}. \end{aligned}$$

#### MFA analysis

**Fig. 3:**
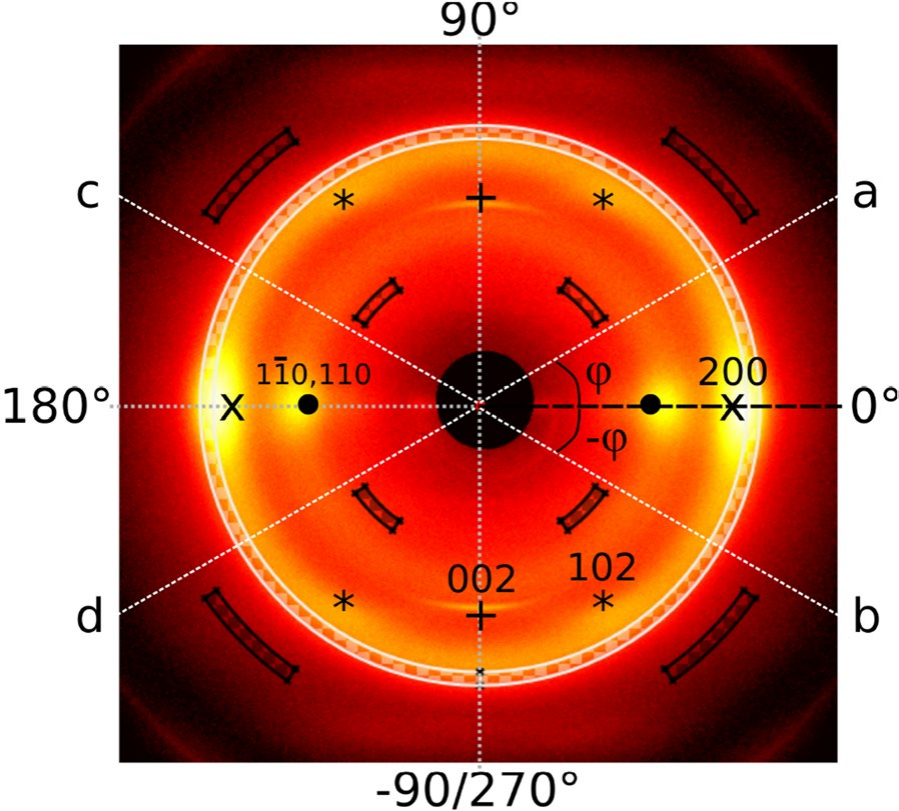
Two-dimensional X-ray scattering pattern of bamboo. Azimuthal angles are defined so that one of the cellulose $\hbox {I}\beta$ 200 diffraction peaks is at 0°. A microfibril angle seen at the azimuthal angle $\phi _{i}$ (*a*) is also seen at the azimuthal angles $-\phi _{i}$ (*b*), 180° $-\phi _{i}$ (*c*), and 180° $+\phi _{i}$ (*d*) due to symmetry. The most notable reflections are annotated and their symmetry is indicated by *symbols*. The regions *highlighted* with a *dark checkerboard* pattern were used to determine background and the region in *light checkerboard* pattern was used to calculate the azimuthal integral used for the microfibril angle analysis. Scattering pattern is of an outer bamboo culm piece measured with set-up 2

In order to subtract the contribution of amorphous components, linear background subtraction was conducted as in [27] from the azimuthally integrated intensities before MFA analysis. Specifically chosen regions with smaller and higher scattering angles than those selected for the 200 reflection region were assumed to contain only the amorphous component (Fig. 3). The regions were selected so that they should not contain any contribution from crystalline diffraction peaks and they were used to approximate a linear background for the 200 reflection region. The background-subtracted intensity was then assumed to contain only the crystalline cellulose component. The 200 reflection region was selected so that the 102 reflection would have minimal contribution to the azimuthal intensities. In general, the observed positions of the cellulose reflections may vary with the measured samples or tissue types. Based on the measured scattering data, the same 200 reflection region could be used for all tissue types in this study.

The mean microfibril angle was calculated as

1 $$\begin{aligned} \langle MFA \rangle = \frac{\int _{-40^{\circ }}^{140^{\circ }}\phi f(\phi ) d\phi }{\int _{-40^{\circ }}^{140^{\circ }}f(\phi ) d\phi }, \end{aligned}$$

where $\phi$ is the microfibril angle and $f(\phi )$ is the MFA distribution^4^. The MFA distribution was obtained by fitting four quadruplets of Gaussian peaks to the azimuthal intensity profile, in addition to a constant. The constant corresponds to a contribution of un-oriented cellulose crystallites and this contribution was not considered when calculating the average MFA. Each Gaussian peak quadruplet maxima were fitted to the angles of $\phi _{i}, -\phi _{i}, {180^{\circ }+\phi _{i}}$ and ${180^{\circ }-\phi _{i}}$ (Fig. 3) based on the model presented in [28]. Only the peaks at $\phi _{i}$ were used to calculate the final MFA distribution and the MFA distribution represents the contribution of all bamboo cell wall layers.

When the observed azimuthal intensity distribution (over 180 degrees) consists of a single narrow and relatively featureless peak, there are no well-established correction procedures for the cell shape factor for cell shapes close to circular. Due to this lack of features in the narrow azimuthal intensity peak, the fitting is more ambiguous and less reliable, especially if too many experimental parameters are fitted. The MFA distribution was therefore determined without a cell shape correction and the average MFA values represent thus the lower limit of the true average MFA values.

In practice, the MFAs are determined with respect to the longitudinal axis of the culm wall. For the parenchyma cells that have an aspect ratio close to one, we define the longitudinal axis of the cell to be parallel to that of the culm wall. With this definition, the observed MFA of ideal, horizontal end caps of the cell would be 90° regardless of the true microfibril orientation within the end cap.

## Results

### X-ray diffraction tomography

**Fig. 4:**
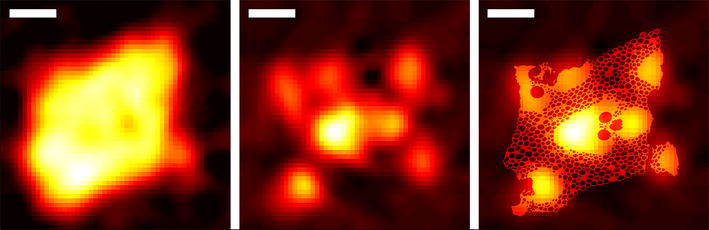
X-ray diffraction tomography. The X-ray diffraction tomography can be used to obtain the cellulose I distribution in 2D (*left*) or the cellulose microfibril orientation (*middle*). The cellulose orientation is plotted on top of the absorption-contrast tomographic reconstruction slice (*right*). The *scale bar* is $400\,\mu \hbox {m}$

The results of the XDT reconstruction for microfibril orientation and cellulose I contrasts are shown in Fig. 4. The cellulose contrast follows clearly the overall shape of the bamboo cross-section and shows the presence of cellulose I, as expected. The microfibril orientation contrast shows clearly that the highest degree of orientation is localized on the fibers and not on the parenchyma.

### Spatially-resolved X-ray scattering

**Fig. 5:**
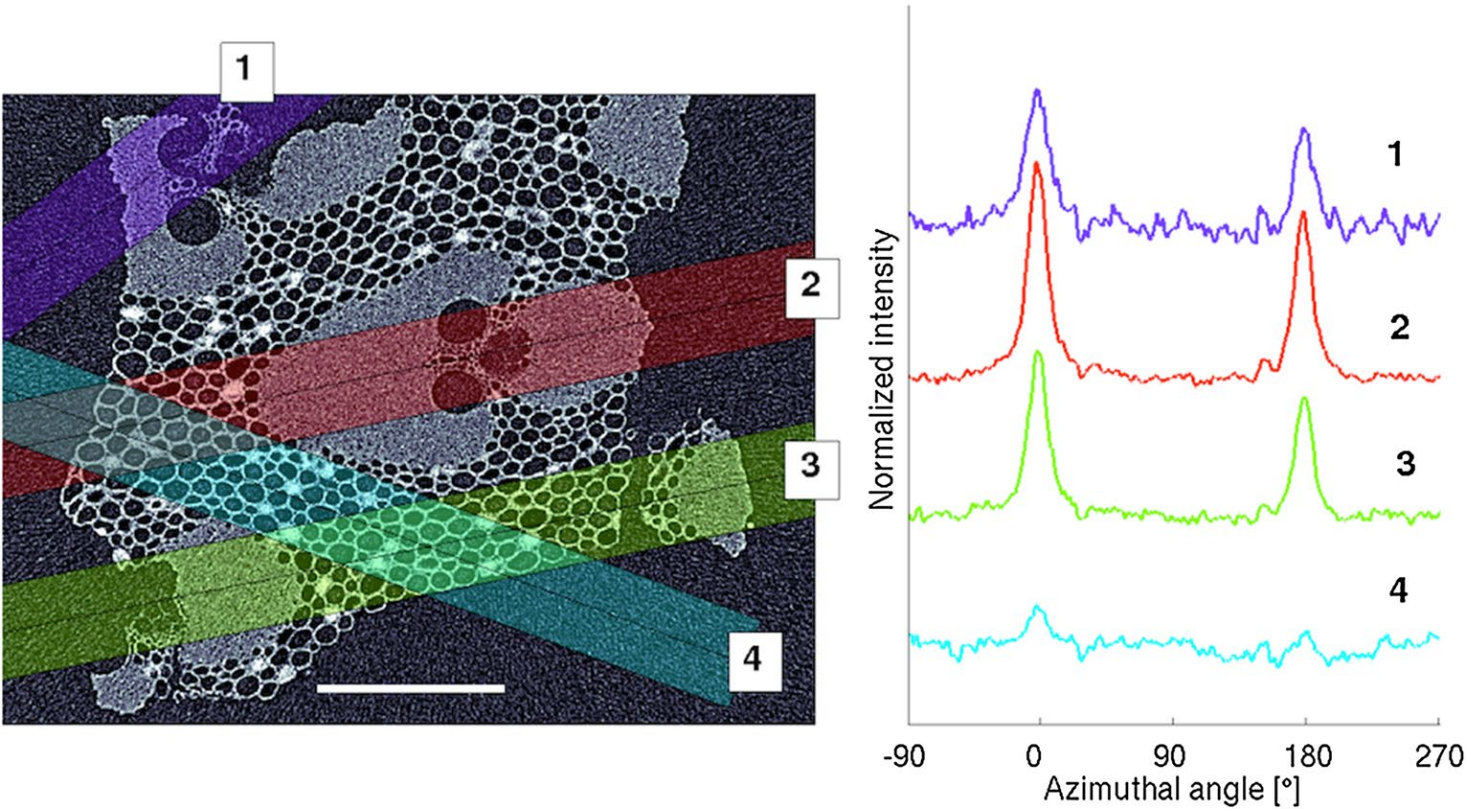
Azimuthal X-ray scattering patterns from a bamboo sample. The spatially-localized X-ray scattering intensities from an outer culm wall bamboo sample shows tissue-specific scattering patterns originating mostly from fibers (1, 2, 3) and from parenchyma only (4). The *rectangles* on the *top* of the tomographic reconstruction slice (*left*,* scale bar* $400\,\mu \hbox {m}$) show the approximate X-ray beam paths for the measurements corresponding to the azimuthal intensity profiles on the* right*. The *scale bar* is $400\,\mu \hbox {m}$ and the profiles are shifted vertically for clarity

**Fig. 6:**
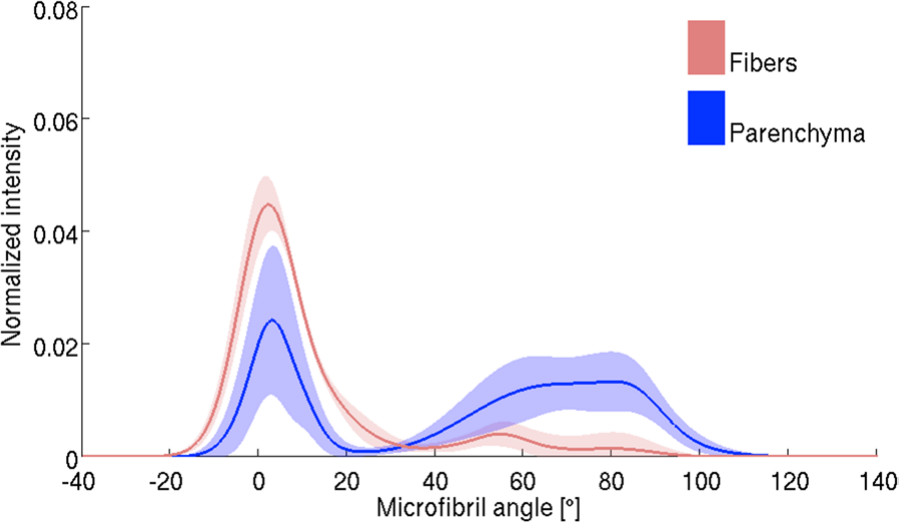
Microfibril angle (MFA) distributions from different tissues. Tissue-selectivity of the method is visualized by plotting the averaged MFA distributions of the fiber- and parenchyma-rich regions of interest. The *shaded area* represents the standard deviation (n = 5)

For each of the five samples measured with both X-ray tomography and X-ray scattering, ROIs were selected as in Fig. 5. The ROI with the highest parenchyma content and the one with the highest fiber content were selected from each sample to calculate the average MFA for the two tissue types. All the fiber-selective measurements (such as measurement 2 of Fig. 5) contain some contribution of parenchyma cells and the parenchyma-selective measurements (measurement 4 of Fig. 5) may contain contribution from neighboring fiber cells. The mean MFA for fiber-selective measurements was $11\pm 8 ^{\circ }$ (n = 5, mean ± standard deviation) and for parenchyma-selective measurements $46\pm 15 ^{\circ }$ (n = 5). The difference between these averages is statistically significant with a *t* test with $p=0.01$. The averaged MFA distributions of the two tissue types are shown in Fig. 6.

**Fig. 7:**
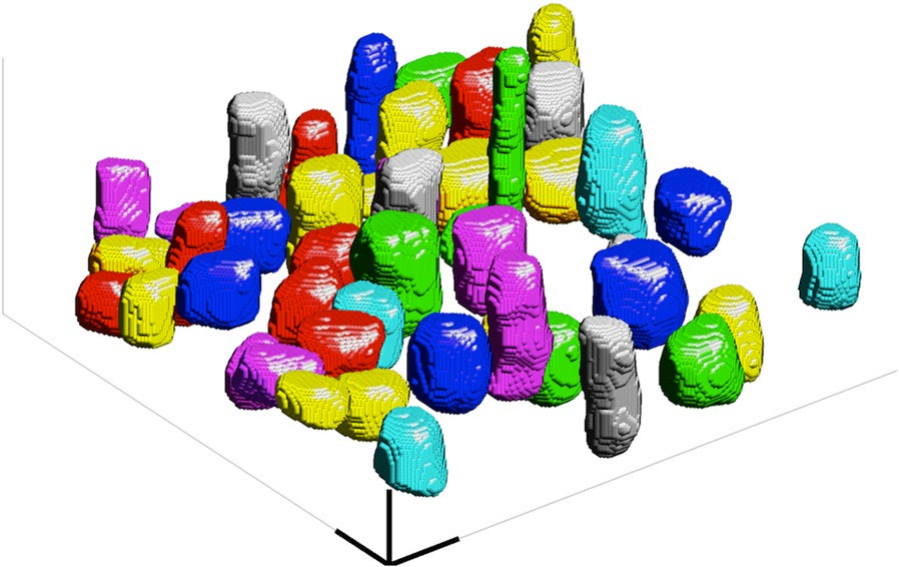
Parenchyma cell lumens. A set of 50 parenchyma cells lumens segmented from the tomographic reconstruction shows that the cells vary greatly in aspect ratio. *Scale bars* ($50\,\mu \hbox {m}$) are shown with *bolded lines*

#### Parenchyma aspect ratio

The parenchyma aspect ratio was calculated from the ROI shown in Fig. 1. An example of the segmented parenchyma cell lumens is shown in Fig. 7. By defining the longitudinal axis of the parenchyma cells to be the longitudinal axis of the culm, aspect ratios below 1 were seen, with an average of 1.6 ± 1.0 (n = 1667, mean ± standard deviation). The parenchyma cell end caps are rather horizontal and due to the low aspect ratio of the cells they have a large contribution to the total scattering intensities. Due to measurement geometry, the scattering contribution of horizontal end caps is seen at MFAs close to 90°.

### Line scan

**Fig. 8:**
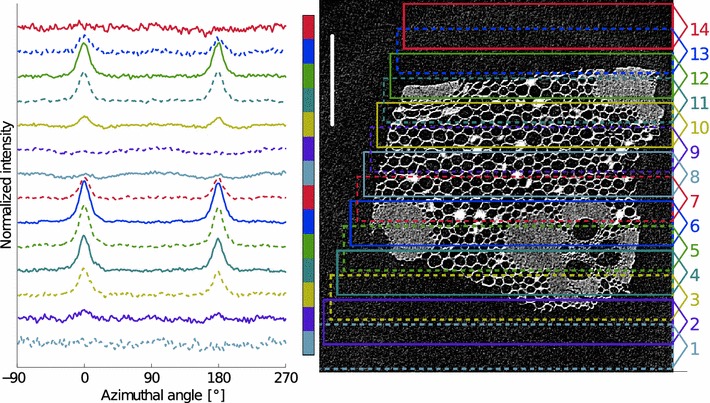
A* line* scan over one sample. A total of 14 scattering measurements were performed by scanning the sample with a 111-$\mu \hbox {m}$ step size. The azimuthal integral over the cellulose $\hbox {I}\beta$ 200-reflection is shown on the left for each measurement (numbered 1–14 from *bottom* to *top*). The *right-hand side* shows the corresponding tomographic reconstruction slice of the inner bamboo culm sample. The *rectangles* overlaid on the *slice* show which part of the sample was sampled for each scattering measurement 1–14. The *scale bar* is $400\,\mu \hbox {m}$

A line scan was performed over one bamboo sample in 111-$\mu \hbox {m}$ steps. A 30-min scattering measurement was performed at each step (Fig. 8). This line scan featured two measurements of parenchyma cells only (#8 and #9), which did not show any preferred orientation parallel to the strong preferred orientation visible in other measurements. The primary orientation of the other measurements can be contributed to fibers in the vascular bundles. The secondary orientation perpendicular to the primary one can be contributed to the parenchyma. Although not easily seen from the azimuthal integrals, most measurements can be expected to contain both primary (fiber) and secondary (parenchyma) orientation.

**Fig. 9:**
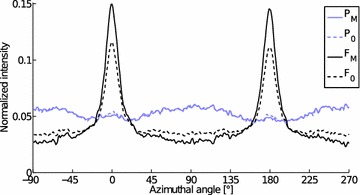
Models of the non-negative matrix factorization (NNMF). The input models (subscript 0) are shown as *dotted curves* and the NNMF results (*subscript M*) are shown with *full lines*. *Black curve* corresponds to the fiber model (F) and the *blue/gray curve* to the parenchyma model (P)

Starting values for the two components for the NNMF fit were selected by summing the line scan measurement data #5 and #6 for the fiber component and #8 and #9 for the parenchyma component. The fiber ratio calculated from the tomographic reconstruction slice for each scattering measurement was also used as the input for NNMF. A good fit for all measurements was obtained ($r^{2}=0.92\pm 0.14$) suggesting that the data can be well explained using the two-component model. The fit yields one component with a sharp orientation peak (fibers) and the other with a broader maxima perpendicular to the first (parenchyma, Fig. 9).

**Fig. 10:**
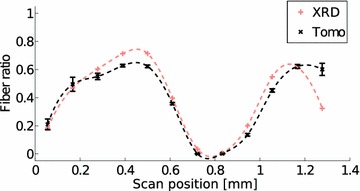
Ratio of fibers by tomography and X-ray diffraction (XRD). Both methods yield similar fiber ratios for the measurements #2 to #13 (Fig. 8). The spline fit shown with a *dotted line* is a guide for the eye

**Fig. 11:**
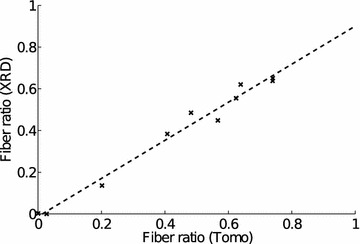
Correlation between tomography fiber ratio (FR) and X-ray diffraction (XRD) FR. FRs of measurements #3 to #12 (Fig. 8) measured with tomography and XRD show excellent linear correlation ($r^{2}=0.96$). The linear component of the fit is 0.91 and the constant is $-0.01$

NNMF yields also weight factors for the two components. The fiber ratio obtained from the LXS data is shown in Fig. 10 as a function of the scan position. The figure also shows the fiber ratio calculated from the tomographic reconstruction slice. A good correlation between these data (Fig. 11, $r^{2}=0.96$) suggests that the two components obtained from the NNMF really are (1) fibers and (2) other cells in the sample, mainly parenchyma. The mean MFAs for these two components are $11\pm 3^{\circ }$ for fibers and $65\pm 10^{\circ }$ for parenchyma.

**Fig. 12:**
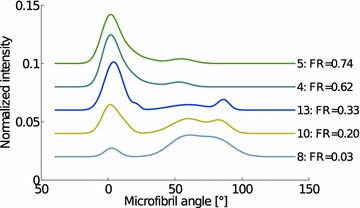
Microfibril angle distributions for representative line scan measurements from Fig. 8 are shown. The *lines* are plotted in order of decreasing X-ray diffraction fiber ratio (FR), from *top* to *bottom* and are shifted vertically for clarity. The number at the end of the *curve* corresponds to the measurement number shown in Fig. 8

**Fig. 13:**
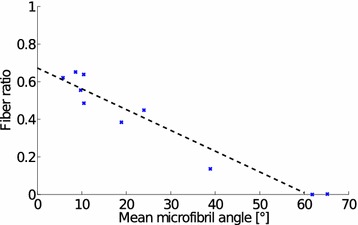
Fiber ratio as function of mean microfibril angle in line scan measurements shows a linear correlation ($r^{2}=0.93$ ) between the parameters. The fiber ratio is calculated from the tomographic reconstruction data

The MFA distributions for representative line scan measurements are shown in Fig. 12. There is a strong linear correlation ($r^{2}=0.93$) between the mean MFA and the fiber ratio determined from tomography (Fig. 13).

### Scattering from average bamboo tissue

**Fig. 14:**
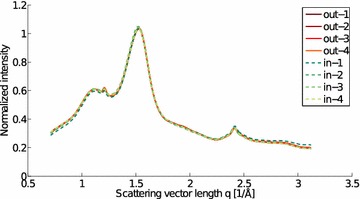
Radial scattering intensities of the Moso bamboo samples. The scattering intensities of the inner (in) and outer (out) culm wall pieces as a function of the scattering vector length are very similar for all measured samples

Full two-dimensional X-ray scattering patterns were obtained with set-up 2 to obtain an average bamboo tissue scattering for inner and outer culm wall samples. In the radially integrated scattering patterns, there are no notable differences between the inner and outer culm wall samples (Fig. 14). This indicates that the relative sample crystallinity^5^ is the same for inner and outer samples. Since the inner samples have significantly smaller fiber ratios, this suggests that there is no substantial crystallinity difference between the fibers and the parenchyma, as has been shown earlier by Abe and Yano [16].

**Fig. 15:**
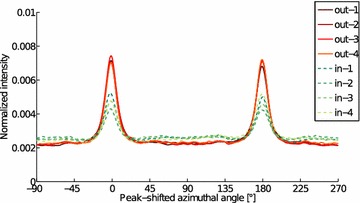
Azimuthal intensities of the Moso bamboo samples. Samples from outer (out) culm show higher degree of orientation than the inner culm samples (in). Amorphous contribution has been subtracted from these curves which represent contribution of crystalline cellulose only

However, there are qualitative and quantitative differences in the microfibril orientation of the inner and outer culm wall samples as evidenced by the azimuthal integrals (Fig. 15). For the outer culm samples, they show an even distribution of MFAs and one sharp orientation peak (primary orientation). The intensity of the primary orientation peak of the inner culm samples varies more and a broad secondary maximum is visible perpendicular to the primary peaks.

The NNMF models obtained from the line scan (Fig. 9) were fitted to the bulk average data of inner and outer culm wall samples to obtain a fiber ratio. A value of $0.35\pm 0.06$ (mean ± standard deviation, n = 4, $r^{2}=0.96\pm 0.02$) was obtained for the inner and $0.68\pm 0.02$ (n = 4, $r^{2}=0.99\pm 0.01$) for the outer samples. A considerably larger fiber ratio for the outer culm wall samples is consistent with literature [5, 14]. The high $r^{2}$ values suggest that the bulk average data can be modeled well using the two-component model obtained from the line scan data.

**Fig. 16:**
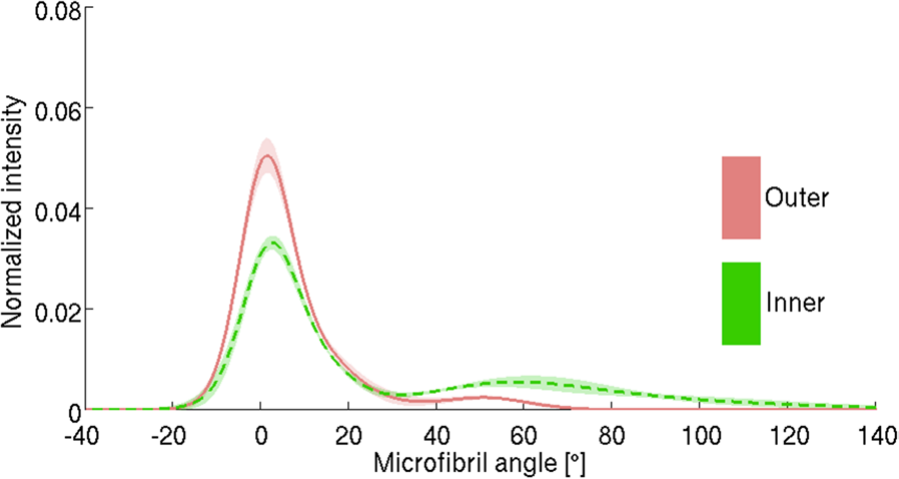
Microfibril angle (MFA) distributions of inner and outer culm wall samples. The mean MFA distribution of the outer culm wall sample is shown with a *solid line* and the inner with a *dotted line*. The *shaded areas* represent the standard deviation (n = 4 for each)

The mean MFA was $27\pm 3 ^{\circ }$ for the inner samples (n = 4) and $7.0\pm 1.4 ^{\circ }$ for the outer samples (n = 4). The mean MFAs of the inner samples are similar to those reported in [11] and the average MFA distributions are shown in Fig. 16.

## Discussion

Spatially-localized WAXS was used to observe tissue-specific MFA orientation in native bamboo culm wall. Unlike previous studies [15, 16], the method presented here is non-destructive and does not require any chemical treatment. The LXS also provided models for different cell types, which can be useful when assessing whether the observed peaks in the MFA orientation distribution originate from different cell types or from different cell wall layers within one cell type. To the authors’ knowledge, no other model has been presented for the parenchyma microfibril orientation distribution based on WAXS data.

The average MFA has considerable influence on the stiffness and strength of wood [29–31] and micromechanical models of wood include it as one of the critical parameters [32–34]. To develop micromechanical models for bamboo, both the highly elongated fiber and the cellular parenchyma tissues, with short aspect ratios, need to be considered, requiring data for the MFA for both. Additionally, the transverse elastic properties of internode bamboo tissue are likely dominated by those of the parenchyma, as those of a unidirectional fiber reinforced composite are dominated by the matrix [35]. Considering the short aspect ratios of the parenchyma cells, the low degree of orientation of the microfibrils in the parenchyma and high degree of orientation of the microfibrils in the fiber (relative to the longitudinal fiber axis), the parenchyma’s role in governing the transverse elastic properties is likely even larger. The tissue specific MFA data obtained by this non-invasive, spatially-localized, WAXS method provides important data needed for understanding the mechanics of bamboo.

The cell shape affects the observed azimuthal integrals of the 200 reflection [36–38]. For rectangular cells, the peak shape can be taken into account by fitting a contribution from all four cell walls [39]. For cells with irregular or circular cross section the correction is more complicated and the 004 reflection has been used for the MFA analysis in some earlier work [37, 40] although it is more contaminated by neighboring reflections [37, 41] than the stronger 200 reflection. In the case of small MFAs, there is more peak overlap of the contributions of different cell wall sides and therefore the fitting is more ambiguous, so the smaller MFAs should be considered to have a rather high external uncertainty. Excluding the shape factor yields a more repeatable fitting process. For circular cells, this method is likely to yield MFAs that are systematically smaller than the true MFAs in the cell wall. Therefore, the average MFA values calculated here should be considered as lower limits.

The variation in peak height of the primary (fiber) orientation in Fig. 15 could be caused by variations in how the different tissue types were sampled (statistical variation). Variation in the degree of orientation as a function of the distance from the inner culm has also been seen earlier [5, 28] and this could also explain the difference seen in Fig. 15 (biological variation). Both factors are likely to contribute to the observed variation in the inner culm wall samples.

The value obtained for the fiber ratio from the tomographic reconstruction slice is sensitive to the binarization parameters and to the reconstruction resolution. Also the tomographic reconstruction resolution used was not sufficient to distinguish individual fiber cells. The fiber volume is therefore overestimated. These fiber ratio values should thus be considered relative values. As such, no comparison was made involving the fiber ratios of different samples. The relative fiber ratio values of one sample can be compared, however, as the factors described do not affect the correlation between the values of individual ROIs from the same sample.

The line scan method could be applied to studying the MFA distribution as a function of radial distance in the bamboo culm wall. This information could be connected to the three-dimensional cell-level structure, obtainable from the microtomography. This includes not only the information on different cell types sampled, but also the information on the cross-sectional cell shapes and the aspect ratios of the cells, both of which affect the observed scattering intensities. Using the spatially-localized line scan method, a better radial resolution and a finer sampling grid could be obtained than has been used in conventional WAXS laboratory methods for bamboo [5, 9, 28].

Set-up 1 is also suitable for measuring diffraction-contrast tomography with a resolution limited mainly by the 200-$\mu \hbox {m}$ beam size. The measured XDT (Fig. 4) showed that the current system can be used to obtain 1D, 2D or even 3D maps with diffraction contrast. Different diffraction-based contrasts can be chosen after the measurements. In addition to the orientation and crystal phase detection presented, impurities, crystallite size or crystallographic parameters can be used for contrast. For example, for samples with more heterogeneous crystalline content than Moso bamboo, e.g. cellulose treated with supercritical water hydrolysis [42, 43], cellulose II content could also be determined.

Some biologically relevant systems that could benefit from in-house XDT are reaction wood, drying of wood, mechanically tested wood and crystallization of biomaterials. The functionalities of all these systems crucially depend on their structural features both at the nanoscale and at the microscale. More generally, the structure and function of all biological and biomimetic materials is determined by the hierarchy of the structure; in biomaterials the structural features cover length scales from atomic to the macroscale and are entangled with the properties of the materials. Using the X-ray techniques presented here, the links and connections between the different length scales can be mapped together in a unique way thus giving pivotal information on the structure-function relationships.

## Conclusions

The applicability of the combined in-house microtomography and diffraction set-up to biologically relevant samples was demonstrated with bamboo samples. The set-up allows tissue-specific X-ray scattering by selecting the region-of-interest from the tomographic reconstruction slice. Further, both a one-dimensional line scan and a two-dimensional diffraction tomography were used to obtain scattering information that can be combined with the three-dimensional X-ray tomography information. The method presented is applicable to a wide range of biological samples and can be performed using a combined bench-top XMT/LXS set-up.

A two-component model was obtained from the azimuthal integrals of the line scan which yielded tissue-specific components of Moso bamboo. The fiber component showed a high degree of orientation, whereas the parenchyma model showed a lower degree of orientation with the preferred orientation perpendicular to that of the bamboo fibers.

Spatially-localized X-ray scattering has been shown to provide novel information on the bamboo culm wall that is only attainable by combining the cellular level information with the nanoscale. LXS thus provides insight on biological materials that is uniquely able to reveal the hierarchical structure in complex biological, or biomimetic, systems.

## Footnotes

1 The commercial Moso bamboo culm material was treated for preservation with a chemical borate treatment before importation. This treatment is not necessary for the X-ray methods and it has little effect on the natural state of the smaller samples cut from the culm wall.

2 A 15 min measurement time was used for one sample.

3 Unlike in [11], due to a multi-layer monochromator, polarization caused by the monochromator was assumed to be naught.

4 A 180-degree sector was used that contained the contribution of all fitted Guassian peaks.

5 Differences in cellulose crystallinities or in texture-independent crystallinities cannot be assessed from our data since only one measurement geometry was used and the cellulose content of the samples was not determined.
